# Degeneration of penicillin production in ethanol-limited chemostat cultivations of *Penicillium chrysogenum*: A systems biology approach

**DOI:** 10.1186/1752-0509-5-132

**Published:** 2011-08-19

**Authors:** Rutger D Douma, Joana M Batista, Kai M Touw, Jan AKW Kiel, Arjen M Krikken, Zheng Zhao, Tânia Veiga, Paul Klaassen, Roel AL Bovenberg, Jean-Marc Daran, Joseph J Heijnen, Walter M van Gulik

**Affiliations:** 1 Department of Biotechnology, Delft University of Technology, Kluyver Centre for Genomics of Industrial Fermentation, Julianalaan 67, 2628 BC Delft, The Netherlands; 2 Molecular Cell Biology, Groningen Biomolecular Sciences and Biotechnology Institute, University of Groningen, P.O. Box 11103, 9700 CC Groningen, The Netherlands; 3 DSM Biotechnology Center, P.O. Box 425, 2600 AK Delft, The Netherlands

## Abstract

**Background:**

In microbial production of non-catabolic products such as antibiotics a loss of production capacity upon long-term cultivation (for example chemostat), a phenomenon called strain degeneration, is often observed. In this study a systems biology approach, monitoring changes from gene to produced flux, was used to study degeneration of penicillin production in a high producing *Penicillium chrysogenum *strain during prolonged ethanol-limited chemostat cultivations.

**Results:**

During these cultivations, the biomass specific penicillin production rate decreased more than 10-fold in less than 22 generations. No evidence was obtained for a decrease of the copy number of the penicillin gene cluster, nor a significant down regulation of the expression of the penicillin biosynthesis genes. However, a strong down regulation of the biosynthesis pathway of cysteine, one of the precursors of penicillin, was observed. Furthermore the protein levels of the penicillin pathway enzymes L-α-(δ-aminoadipyl)-L-α-cystenyl-D-α-valine synthetase (ACVS) and isopenicillin-N synthase (IPNS), decreased significantly. Re-cultivation of fully degenerated cells in unlimited batch culture and subsequent C-limited chemostats did only result in a slight recovery of penicillin production.

**Conclusions:**

Our findings indicate that the observed degeneration is attributed to a significant decrease of the levels of the first two enzymes of the penicillin biosynthesis pathway, ACVS and IPNS. This decrease is not caused by genetic instability of the penicillin amplicon, neither by down regulation of the penicillin biosynthesis pathway. Furthermore no indications were obtained for degradation of these enzymes as a result of autophagy. Possible causes for the decreased enzyme levels could be a decrease of the translation efficiency of ACVS and IPNS during degeneration, or the presence of a culture variant impaired in the biosynthesis of functional proteins of these enzymes, which outcompeted the high producing part of the population.

## Background

The rate and yield of penicillin production by the fungus *Penicillium chrysogenum *have been increased many-fold after its discovery by Alexander Fleming in 1928 [1]. Initially this was performed by successive rounds of random mutagenesis and selection [2-4]. Recently, the genome sequence was elucidated [5], facilitating transcriptome [6] and proteome studies [7,8]. In combination with flux calculations and metabolome studies [9-11] this enables a systems biology analysis to identify metabolic engineering targets to enhance penicillin production.

Although in current industrial production strains the biomass specific penicillin production rate (q_p_) has increased many fold, *P. chrysogenum *does not maintain its high production capacity in extended fermentations. As early as 1932 the loss of production capacity was reported upon continued sub cultivation of penicillin producing cultures of *P. chrysogenum *[12] and also strains further on in the lineage were reported to loose penicillin productivity in prolonged carbon limited chemostat fermentations [9,13-17]. Degeneration of product formation has not only been observed for penicillin-G (PenG) production, but also for other antibiotics, e.g. adipoyl-7-aminodeacetoxycephalosporanic (ad-7-ADCA) acid production in *P. chrysogenum *[18], oxytetracycline production in *Streptomyces rimosus *[19] and tylosin production in *Streptomyces fradiae *[20]. This generally observed (partial) loss of production capacity during extended cultivation is usually referred to as degeneration. This phenomenon is of major relevance because it prevents the application of a continuous production process, which, for bulk production of antibiotics such as penicillin, would be economically more favorable compared to batch or fed-batch fermentations, because of less down time and thus more efficient use of equipment.

It is easily understood that if a micro-organism (partially) loses its production capacity, more substrate and energy can be used for growth. Penicillin production requires a significant amount of carbon and metabolic energy [16] giving non-producing cells a competitive advantage. A strain which can keep its high productivity for a longer time (absence of degeneration) would result in a more productive fermentation process, and would allow penicillin fermentations to run in continuous mode instead of the now applied fed-batch mode.

**Figure 1:**
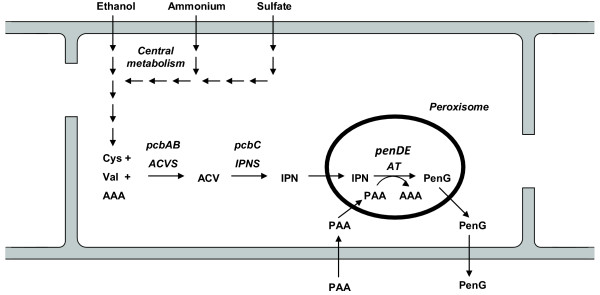
**Penicillin pathway in *P. chrysogenum***. L-cysteine (Cys), L-valine (Val) and α-amino adipic acid (AAA) are produced from ethanol in central metabolism. These three precursor amino acids are converted into L-α-(δ-aminoadipyl)-L-α-cystenyl-D-α-valine (ACV) by the enzyme L-α-(δ-aminoadipyl)-L-α-cystenyl-D-α-valine synthetase (ACVS, which is coded for by the gene *pcbAB*). ACV is subsequently converted to isopenicillin-N (IPN) by the enzyme isopenicillin-N synthase (IPNS, which is coded for by the gene *pcbC*). IPN is then transported into the peroxisome where it is converted into PenG with the precursor PAA, which is added to the medium and imported in the cell and then activated by phenylacetyl CoA ligase to PAA-CoA which is used by the enzyme acyl coenzyme A: Isopenicillin N acyltransferase (AT, which is coded for by the gene *penDE*). The product PenG is then transported out of the peroxisome and out of the cell into the cultivation medium.

In this study, different -omics techniques were simultaneously applied in an attempt to obtain a systems understanding of degeneration of production of PenG in *P. chrysogenum *during prolonged carbon limited chemostat cultivations. This system study focuses mainly on the penicillin pathway (figure 1), in an industrial high penicillin producing strain, *P. chrysogenum *DS17690. Prolonged chemostat cultivations up to 600 hours (30 generations) with ethanol as the sole limiting carbon source were performed, because degeneration was observed to be more pronounced with ethanol as growth limiting substrate, compared to glucose [9]. The analysis included determining the number of penicillin gene clusters, genome-wide transcriptome analysis, measurements of the protein levels of penicillin pathway enzymes, quantification of the number of peroxisomes (microbodies where the last step of the penicillin pathway is located), metabolome analysis (of central metabolism, nucleotides, penicillin pathway intermediates including intracellular phenylacetic acid (PAA) and PenG) and metabolic flux calculations.

## Results

### General observations

To study the degeneration of penicillin production, four replicate chemostat cultivations of *P. chrysogenum *DS17690 were performed with ethanol as the sole limiting carbon source. All chemostat cultivations were preceded by a batch phase during which the culture grew exponentially until all ethanol was depleted. During the batch phase no penicillin was produced. This has also been observed during batch growth on glucose as carbon source [21,22] and is generally attributed to carbon catabolite repression. Apparently also non-limiting concentrations of ethanol result in repression of penicillin production. Using TOC measurements to quantify non-identified by-product formation (e.g. proteins/peptides and polysaccharides, partly as a result of cell lysis), the carbon and redox balances were found to close satisfactory (100 ± 5%) for all chemostat runs, showing negligible (<10%) by-product formation. Taking the conversion of PAA to *ortho*-hydroxyphenyl acetic acid (o-OH-PAA) into account, which in our cultures accounted for approximately 10% of the total PAA consumption, the PAA balance closed between 90 - 110%, indicating the absence of PAA catabolism, which is in agreement with earlier observations [23].

**Figure 2:**
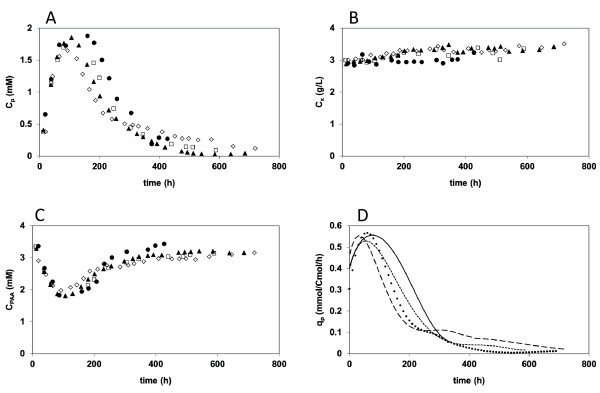
**Time patterns of the concentrations of biomass, PenG, PAA and the biomass specific PenG production rate q_p _during ethanol limited chemostat cultivation**. (A) PenG, (B) biomass and (C) PAA concentrations in chemostat 1 (filled circles), 2 (open squares), 3 (filled triangles) and 4 (diamonds) and (D) biomass specific PenG production in chemostat 1 (solid line), 2 (small stripes), 3 (dots) and 4 (large stripes).

The measured time patterns of the concentrations of biomass, PenG, PAA and the biomass specific PenG production rate q_p _are shown in figure 2. It can be seen from these time patterns that the chemostat cultivations were well reproducible. Figure 2D shows that the biomass specific penicillin production rate (q_p_) reached a maximum value at about 40-80 hours after the start of the chemostat phase, which is the time needed for the penicillin pathway enzymes, which are repressed during batch cultivation, to reach their maximum levels [17]. However, directly after reaching a maximum value degeneration of penicillin production was observed to set in, as can be inferred from the decrease of the PenG concentration and a corresponding increase of the residual PAA concentration. The decrease of the penicillin production coincided with a gradual increase of the biomass concentration. This has been observed before [9,13,15] and is not unexpected because a decrease of penicillin production results in an increased availability of substrate for growth. Considering the estimated ATP stoichiometry of this *P. chrysogenum *strain [16], the decrease in penicillin production appeared to be in stoichiometric correspondence with the increase in biomass concentration. Although the pattern of the degeneration profile was highly reproducible, as can be seen from figure 2, the profiles were slightly shifted in time for the individual cultivations. During degeneration the biomass specific penicillin production rate, q_p_, decreased on average about 10-fold.

### Flux analysis

**Figure 3:**
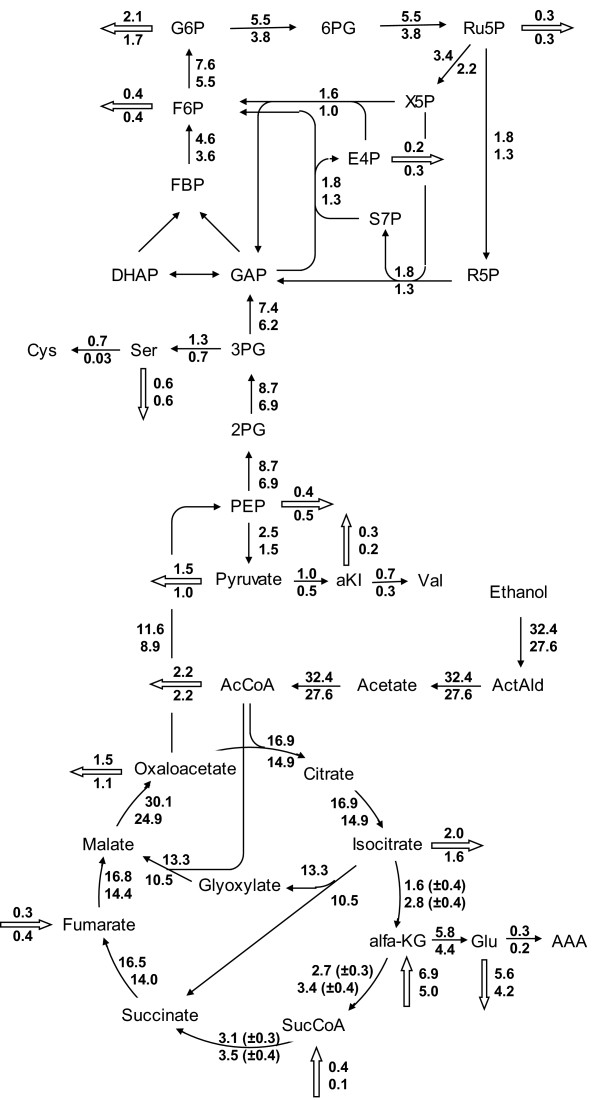
**Calculated flux distribution in the central metabolism of *P. chrysogenum*, assuming pseudo steady-state**. The numbers next to the arrows represent the average flux of chemostat 3 and 4 in mmol/Cmol/h at the penicillin production peak (t = 75 h, upper number) and the degenerated culture (t = 500 h, lower number). The direction of the arrow indicates the direction of the flux. Not all fluxes in the network are shown. The standard errors of the fluxes shown are smaller than 5% unless indicated otherwise.

Metabolic flux analysis, using the stoichiometric model for growth and PenG production of van Gulik et al. [9], was applied to estimate the flux distributions during maximum penicillin production (t = 75 h) and after degeneration (t = 500 h). The fluxes through central carbon metabolism and towards the biosynthesis of the penicillin precursors alpha aminoadipic acid (AAA), cysteine (Cys) and valine (Val) for these two conditions are shown as a flux diagram in figure 3. By far the biggest change in flux was observed for cysteine biosynthesis. Because under high producing conditions almost all produced cysteine is used for PenG biosynthesis and associated by-products (e.g. 6-aminopenicillanic acid (6-APA) and 8-hydroxypenicillic acid (8-HPA)), the degenerated culture had an approximately 20-fold reduced flux in the pathway towards cysteine. Although less drastic, the biosynthetic flux towards valine (Val) decreased about 3-fold, while the flux towards AAA decreased with 50%. Although PenG biosynthesis does not result in net consumption of AAA, it is partly converted to the by-product 6-oxopiperidine-2-carboxylic acid (OPC) which is excreted. During degeneration the formation of OPC decreased steeply (additional file 1, figure S15), resulting in a reduced flux towards AAA. Because penicillin production is assumed to require reducing equivalents in the form of NADPH (mainly for sulphate reduction to synthesize cysteine) the calculated flux through the pentose phosphate pathway is significantly reduced in the degenerated culture. Although in this case the outcome of these MFA calculations depends on the cofactor specificities in the stoichiometric model, it has indeed been shown, i.e. from ^13^C based gluconate tracer studies, that Penicillin production puts a major burden on the supply of cytosolic NADPH [24,25].

### Copy number of the penicillin gene cluster

**Figure 4:**
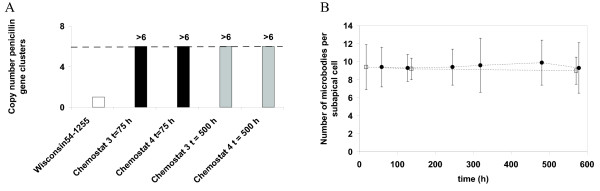
**Results of the penicillin gene cluster and microbody quantifications**. (A) Copy number of the penicillin gene cluster in the reference strain (Wisconsin 54-1255, 1 penicillin gene cluster, white bar) and in two prolonged chemostat runs of *P. chrysogenum *DS17690 at the penicillin production peak (t = 75 h, black bars) and the degenerated state (t = 500 h, grey bars). (B) Average number of microbodies per sub apical cell during chemostat cultivation: (filled circles) chemostat 5, (open squares) chemostat 6.

The penicillin production pathway genes (figure 1) are incorporated as a cluster in the genome [26]. The increased penicillin production of *P. chrysogenum *DS17690 is partly caused by the fact that this strain contains six to seven copies [5] of the penicillin gene cluster compared to the parent strain *P. chrysogenum *NRRL1951, which contains only a single copy [4]. A plausible hypothesis for the loss of penicillin production would be that the loss of production capacity is caused by the gradual loss of penicillin gene clusters, as has been observed by Christensen et al. [15] for penicillin-V (PenV) synthesis in a different industrial *P. chrysogenum *strain. Therefore the copy number of the penicillin gene cluster was quantified during degeneration, using real time PCR. This quantification method yields accurate results up to a number of 6 penicillin gene clusters. Figure 4A shows the result for two time points, namely t = 75 h (maximum q_p_) and t = 500 h (degenerated, low q_p_) for 2 independent chemostat cultures (chemostats 3 and 4). It can be seen from this figure that both during maximum penicillin production and after degeneration of the chemostat culture, more than 6 penicillin gene clusters are present. Therefore this analysis did not provide any indication that degeneration is caused by a loss of penicillin gene clusters.

### Microbody quantification

*P. chrysogenum *is a eukaryotic, compartmentalized microorganism in which the last step of the penicillin production pathway, the acyl transferase (AT) is known to be located in the peroxisomes (see figure 1). Kiel et al., [27] have reported that overproduction of Pc-Pex11p in *P. chrysogenum *Wis54-1255 results in a higher abundance of peroxisomes and a twofold higher penicillin production. Although in the study of Kiel et al. a different strain with a much lower productivity was used, a possible hypothesis for degeneration of penicillin production in our high producing strain, *P. chrysogenum *DS17690, could be a decrease of the number of peroxisomes in time during prolonged carbon limited chemostat cultivation. To investigate if degeneration in our DS17690 strain coincided with a decrease of the number of peroxisomes, similar prolonged ethanol limited chemostat cultivations were carried out with a peroxisome-targeted GFP·SKL producing mutant of *P. chrysogenum *DS17690 [28].

It was found that this derived strain behaved the same as the DS17690 strain, with respect to the maximum penicillin production rate, the steady state biomass concentration and the degeneration profile (data not shown). From the determination of the number of peroxisomes per sub apical cell during degeneration, no evidence was obtained for a decrease (see figure 4B). Nevertheless the specific penicillin production rate of the GFP·SKL strain was observed to have decreased 10-fold, following a similar pattern as was observed for *P. chrysogenum *DS17690.

### Transcriptome analysis

To investigate whether the observed degeneration is related to changes in the expression of genes of the penicillin biosynthesis pathway and/or connected metabolic routes, a transcriptome analysis was performed on two sets of four samples which were taken throughout a duplicate chemostat run (chemostats 1 and 2). The data were treated as a time series experiments in duplicate. The average coefficient of variation of the transcriptome data derived from duplicate degeneration experiments did not exceed 25%. The level of the actA and gdh2 genes that are commonly used loading standards for Northern analysis varied by less than 21%. Firstly, the results were tested for the hypothesis that in this time series of samples no changes in the level of the transcripts would occur. It was found that of the 14000 transcripts in *P. chrysogenum *about 1000 showed significant differential expression throughout the prolonged chemostat cultures.

**Figure 5:**
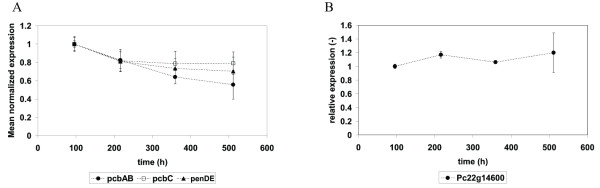
**Mean normalized expression of genes throughout ethanol limited chemostat cultivation. The numbers are averages for chemostats 1 and 2**. (A) The penicillin pathway genes *pcbAB *(ACVS, filled circles), *pcbC *(IPNS, open squares) and *penDE *(AT, filled triangles). (B) The gene Pc22g14600 (filled circles).

Clearly, changes in expression of the three penicillin biosynthesis genes are of prime interest. It was indeed observed that the expression levels of all genes present in the penicillin gene cluster decreased (figure 5A). However, only the almost twofold decrease in *pcbAB *(ACVS) appeared significant, while this was not the case for the smaller decrease in *pcbC *(IPNS) and *penDE *(acyl coenzyme A: Isopenicillin N acyltransferase, AT). The expression level of phenylacetyl CoA ligase (Pc22g14900) did not decrease. These results agree with the observation that there is no significant loss of the copy number of penicillin gene clusters. Nevertheless, global down regulation of secondary metabolism could have resulted in lower transcript levels of the penicillin biosynthesis genes. However, the transcript levels of PcVelA (Pc13g13200) and PcLaeA (Pc16g14010), two major homologues of the velvet complex and know regulators of penicillin biosynthesis [29,30] did not show any change during degeneration.

Apart from the biosynthesis of a product, a sufficient capacity for the import of precursors and the export of the product is crucial. In a genome-wide expression study with *P. chrysogenum *carried out under PenG producing and non-producing conditions, a putative gene for PAA export (Pc22g14600) was identified [6]. The expression level of this gene during the degeneration experiment is depicted in figure 5B, showing no change during the prolonged chemostat run.

**Figure 6:**
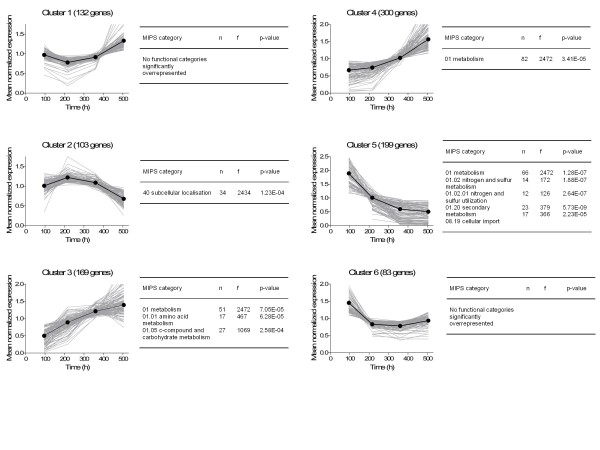
**The 6 different transcriptome clusters in which the differentially expressed genes were divided and subjected to a functional category analysis**.

Changes in expression levels of other genes might give additional indications about the cause of degeneration of penicillin production. Therefore the 1000 differentially expressed genes were grouped into six different clusters based on the trend of their behavior as can be seen in figure 6. Subsequently, a functional category analysis was made within the six clusters. Most interesting observations were made in the functional categories in cluster 5, the one in which the genes are continuously down regulated throughout the experiment. Mainly nitrogen and sulfur metabolism and utilization genes were down regulated throughout the prolonged chemostat cultivations. Further analysis revealed that many of the down regulated genes are related to the biosynthesis pathway of cysteine, one of the precursors of the penicillin pathway (additional file 1, figure S4).

**Figure 7:**
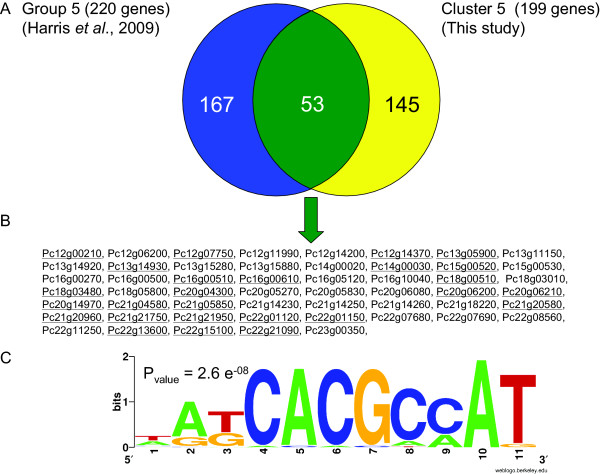
**Genes of which the expression is positively correlated with PenG production**. (A) Venn diagram of the genes comprising cluster 5 and of the genes of which the transcript level was reported to be specifically higher under PenG producing conditions (group 5 in Harris et al. [6]). (B) the 53 genes that are overlapping the two sets. The genes underlined contained at least one cis-regulatory motif, as indicated in (C), in their promoter sequences (promoter sequences includes the region -900, -1). (C) overrepresented cis-regulatory motif in the set of the 53 genes. The motif has been identified using the MEME software and the logo has been edited using the Weblogo software.

An interesting observation was made in the comparison between the down regulated 145 genes in cluster 5 and the 167 genes which were only up regulated under PenG producing conditions, as reported by Harris et al. [6]. As shown in figure 7A and 7B, 53 genes occur in both groups, meaning that these genes are positively associated with PenG production. Using the MEME software [6,31] an overrepresented *cis*-regulatory motif was found in the 900 base pairs upstream of the mentioned 53 genes (figure 7C). This implies that this group of genes might be co regulated.

An analysis of the expression levels of gluconeogenesis and TCA cycle genes shows that the transcript levels of central metabolism did not significantly change, which is not surprising because of the small flux change as a result of degeneration.

### Protein levels

**Figure 8:**
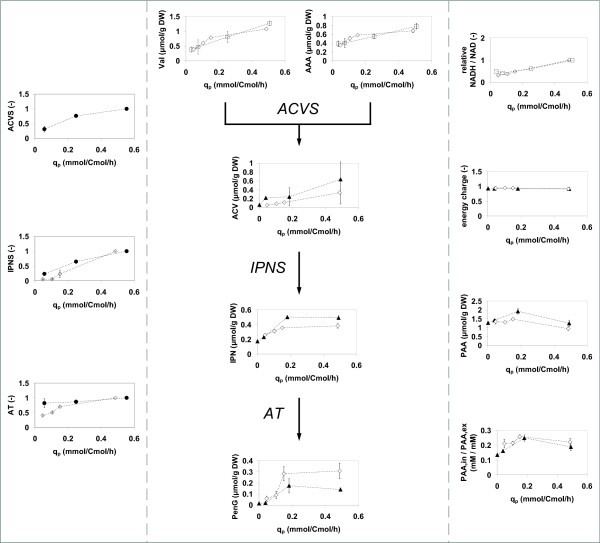
**Levels of proteins and metabolites associated with PenG production, plotted against the specific penicillin production q_P_**. Left panel: Relative protein amounts of the enzymes of the penicillin biosynthesis pathway (see Figure 1) compared to the reference condition at the penicillin production peak (first time point) in chemostat 1 (filled circles) and chemostat 4 (open diamonds). Middle panel: Intracellular levels of penicillin precursors and intermediates in chemostat 2 (open squares), chemostat 3 (filled triangles) and chemostat 4 (open diamonds). Right panel: Energy charge, calculated ratio of NADH/NAD from the Mannitol6P/F6P sensor reaction, intracellular PAA and the concentration ratio of PAA in chemostat 2 (open squares), chemostat 3 (filled triangles) and chemostat 4 (open diamonds).

To investigate whether changes in the levels of the penicillin biosynthesis pathway enzymes ACVS, IPNS and AT could be responsible for the strongly reduced PenG production, these were analyzed via Western blotting for chemostats 1 and 4. Indeed, the levels of ACVS, IPNS and, to a lesser extent, AT were found to decrease during degeneration. Figure 8 (left panel) shows how these changes correlate with the decrease in q_p_. Note that a low q_p _corresponds to the degenerated condition. The ACVS level could only be measured for chemostat 1, and shows an approximate 3-fold decrease after about 400 h of cultivation for both chemostats analyzed. The change of the IPNS level was more pronounced, and decreased 5 to 20-fold, for chemostat 1 and 4 respectively. The amount of AT was found to have decreased much less. Figure 8 (left panel) shows that q_p _is proportional to the amount of IPNS and not to the amount of AT, which is in agreement with earlier findings [17].

### Metabolome analysis

Intracellular levels of 17 central carbon metabolism intermediates, 16 free amino acids, 9 penicillin biosynthesis related metabolites and the adenine nucleotides were monitored throughout the chemostat cultivations (additional file 1, figures S5-14). The majority of the central metabolites did not show large changes during degeneration. However, the levels of several intermediates of gluconeogenesis were lowest at the time the first sample was taken (about 100 h of chemostat cultivation) and reached a stable and somewhat increased value thereafter. The intracellular free amino acid levels did not change during degeneration, with the exception of the β-lactam precursors Val and AAA, which decreased consistently and reached a respectively 2.5 and 2 fold lower level at the end of the cultivation. Unfortunately the cysteine level appeared too low to be measured accurately. Significant changes were observed for the penicillin pathway related metabolites (figure 8). In this figure the levels of these metabolites are plotted as a function of the penicillin production rate. Similar to the decrease of the levels of the direct precursors Val and AAA, the ACV level decreased consistently with the decreasing penicillin production rate. Also the intracellular levels of IPN and PenG decreased, but only for penicillin production rates below 0.2 mmol/Cmol/h. The decrease of the penicillin production rate and corresponding decreases in the intracellular levels of the precursors and intermediates of the pathway during degeneration was also clearly reflected in strongly decreased extracellular levels of related by-products, i.e. the carboxylated form of AAA, 6-oxo-piperidine-2-carboxylic acid (OPC), 6-aminopenicillanic acid (6-APA), 8-hydroxypenillic acid (8-HPA) and penicilloic acid (PIO).

### Continued sub cultivation

**Figure 9:**
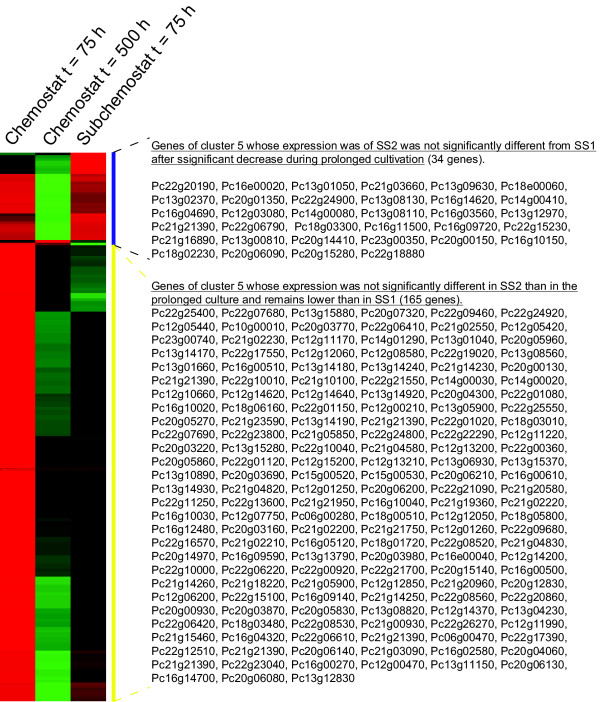
**Heat map of the transcript level of the genes comprising cluster 5**. The heat map displays the mean normalized data at the penicillin production peak (t = 75 h), of the degenerated culture (t = 500 h) and during the steady state of the continued sub cultivation (t = 75 h) obtained by inoculation of a new chemostat with biomass of the degenerated culture.

To verify to which extent degeneration is a reversible process, the degenerated culture of chemostat 4 was used as the inoculum for two second chemostat cultures, named sub-chemostats 4.1 and 4.2. After the subcultures reached a steady state, the same analyses were performed as for the parent culture. No differences were observed with respect to the copy number of the penicillin gene cluster, the protein levels of the penicillin biosynthesis pathway enzymes and the metabolite levels, compared with the parent culture at the time of inoculation of the continued sub cultivation (additional file 1, figures S2, S3, S5-14). The same holds for the transcript levels (see Figure 9). Most genes which were down regulated throughout degeneration of penicillin production, remained down regulated during sub cultivation. Interestingly, the penicillin production rates of the two sub cultivations were 2-fold higher compared to the degenerated parent culture. Apparently the loss of penicillin production is partly reversible after a period of cultivation under carbon excess conditions, which lasted in this case for about ten generations. Remarkably, the specific growth rate measured during the batch phase of the continued sub cultivation was 25% higher than that of the batch phase of the initial cultures (see Table 1). This coincided with an up regulation of the putative gene encoding for alcohol dehydrogenase (Pc21g22820) during chemostat cultivation, preceding the regrowth in batch culture.

**Table 1:** Maximum growth rate and biomass specific penicillin production rate in 4 chemostats and 2 sub chemostats inoculated with degenerated culture of chemostat 4

Chemostat run	μ_max _in batch phase (h^-1^)	q_p,max _in chemostat phase (mmol Cmol^-1 ^h^-1^)	q_p _at time of continued sub cultivation (mmol Cmol^-1 ^h^-1^)
Chemostat 1	0.088	0.52	-
Chemostat 2	0.086	0.52	-
Chemostat 3	N.D.	0.52	-
Chemostat 4	0.089	0.54	-
Sub chemostat 4.1	0.109	0.11	0.04
Sub chemostat 4.2	0.112	0.10	0.04

## Discussion

Using a systems biology approach, we attempted to obtain a better understanding of the well-known but poorly understood phenomenon of strain degeneration in antibiotics production, whereby we used penicillin-G production in a high producing, former industrial strain of *P. chrysogenum *as a model system. Therefore the strain was grown in carbon limited chemostat cultures, with ethanol as sole carbon source. In the four replicate cultivations we performed, the biomass specific penicillin production rate, q_P_, followed a characteristic and reproducible time pattern. During the first 40 - 80 hours of carbon limited chemostat cultivation the productivity increased to a maximum level of approximately 0.55 mmol/Cmol/h. (see Figure 2D). Thereafter the productivity decreased steeply to reach a less than tenfold reduced level. Reaching a tenfold reduced productivity took about 270 hours for three of the four cultivations (which corresponds with 11-12 generations) and more than 500 hours (22 generations) for one culture, showing that degeneration of penicillin production is a relatively fast and reproducible process. The fact that a more than tenfold decrease in penicillin production occurred within about 12 generations makes it very unlikely that it is caused by random mutation events.

It is known that in high producing strains of *P. chrysogenum* the copies of the gene cluster containing the penicillin biosynthesis genes [32,33] are present as tandem repeats [2,26]. It has been suggested that the amplification of the penicillin gene cluster during the strain improvement process of random mutagenesis and selection has occurred through a process of chromatid misalignment and recombination, facilitated by recombinogenic regions. This mechanism could also induce deletions of penicillin gene clusters [2]. Especially during long term cultivation of a high copy number strain under conditions where penicillin production is a disadvantage rather than an advantage, e.g. in carbon limited chemostat cultures, it can be expected that the strain gradually loses its production capacity through the loss of penicillin gene clusters. This was clearly not the case in our cultures, which showed a rapid, more than tenfold loss of penicillin producing capacity, without a detectable decrease of the copy number of the penicillin gene cluster.

Alternatively, the observed decrease in penicillin production could have been caused by global (down) regulation. It has been shown that two major homologues of the velvet complex, PcVelA and PcLaeA, which play a role as global regulators of secondary metabolism, also control penicillin biosynthesis in *P. chrysogenum *[29,30]. However, no significant changes in transcript levels were observed for PcVelA and PcLaeA during degeneration (approximately 12% variation for both transcripts). The absence of transcriptional regulation is further confirmed by the observation that the transcript levels for the key enzymes of the pathway (ACVS, IPNS, AT, phenylacetyl coA ligase) show little or no change. Nevertheless, a strong down regulation of sulfur and nitrogen metabolism was observed which coincided with a decrease of the intracellular levels of Val and AAA, with a factor 2.5 and 2 respectively. Assuming a cellular volume of 2.5 mL per g DW the intracellular Val concentration decreased from approximately 400 to 160 μM while the AAA concentration decreased from 320 to 160 μM during degeneration. These values are still well above the in-vitro determined K_m _values of ACVS for Val and AAA of 80 μM and 45 μM respectively [34]. It seems therefore unlikely that the observed decrease of the intracellular levels of Val and AAA are responsible for the 10 fold decreased penicillin production rate. The down regulation of sulfur and nitrogen metabolism and the concomitant decrease of the intracellular levels of Val and AAA levels could also be a result of the decreased need for penicillin precursors rather than a cause for the decreased production. It has been found for a derivative of the *P. chrysogenum *strain used in this study, from which all copies of the penicillin gene cluster were deleted, that nitrogen and sulfur metabolism were also strongly down regulated in comparison with the producing strain [6].

The penicillin pathway flux is in principle dependent on the capacities of the enzymes and transport steps in the pathway and the levels of the amino acid precursors, as discussed above. However, the biosynthesis of penicillin requires also reducing equivalents (i.e. in the form of NADPH) and energy (ATP) [9]. The decreased penicillin production could therefore also be caused by a decreased availability of metabolic energy, the more since PenG production has been reported to be associated with a large additional consumption of ATP [16]. However, the intracellular levels of adenine nucleotides and the energy charge before degeneration were similar as found previously [35] and their values remained stable while the penicillin production decreased significantly (figure 8, upper right panel). It is therefore not very likely that a decreased ATP availability could have been the reason for the decreased penicillin production. To study possible effects of a changed redox supply, information on the NAD(P)H to NAD(P) ratio is required. The ratio of Mannitol6P to F6P can be used as a sensor reaction for the cytosolic NADH to NAD ratio [36]. The thus calculated relative NADH to NAD ratio (figure 8, right panel) indeed decreased with decreasing q_p_, indicating a more oxidized cytosol in the degenerated culture. In a more oxidized cytosol the availability of NADPH might also decrease, thus negatively influencing the ratio between L-α-(δ-aminoadipyl)-L-α-cystenyl-D-α-valine (ACV) and bis-ACV [37], which could decrease the availability of ACV and result in a decreased flux through the penicillin pathway. The results from the other redox-couple, FBP and G3P pointed to similar changes in the NADH to NAD ratio. Finally, in the completely degenerated culture the intracellular levels of the pathway intermediates (ACV, isopenicillin-N (IPN)) and PenG were significantly decreased. The best way to interpret the (complex) relation between the pathway flux and changes in metabolite levels of these is by constructing a complete kinetic model of the pathway, in which changes in protein, redox and metabolite levels are taken into account.

The last step of the penicillin production pathway is known to be located in the peroxisomes (see figure 1). Kiel et al., [27] have reported that overproduction of Pc-Pex11p in *P. chrysogenum *Wis54-1255 results in a higher abundance of peroxisomes and a twofold higher penicillin production. However, no change in peroxisome number was observed during degeneration of our *P. chrysogenum *strain, which was confirmed by the absence of any significant changes in transcript levels of the corresponding PEX genes. Furthermore, if a decrease in peroxisome number would lead to decreased penicillin production this must be through a decrease of the capacity of the last step of the pathway, i.e. AT. This should result in a corresponding decrease of the measured AT level. However, the AT level did not decrease significantly during degeneration. Furthermore, if a decrease of the in-vivo capacity of AT would have been the cause of the decreased productivity, this would have resulted in an accumulation of the pathway intermediates ACV and IPN, which was also not observed.

Recently, Bartoszewska et al. [38] showed that deletion of the *P. chrysogenum *ortholog of the *Saccharomyces cerevisiae *serine-threonine kinase, atg1, resulted in impairment of autophagy in *P. chrysogenum*. They also observed that atg1 deletion resulted in an increase in the enzyme levels of the penicillin biosynthetic pathway and enhanced penicillin production. Autophagy could have played a role in the observed degeneration of pencillin production in our carbon limited chemostat experiments, because in filamentous fungi autophagy has been shown to be involved in nutrient recycling under starvation conditions [39].

We indeed observed that the protein levels of ACVS and IPNS decreased three, respectively five- to twentyfold during degeneration. However, re-cultivation under unlimited (batch) conditions did not result in a recovery of the levels of ACVS and IPNS and only a very limited increase in PenG production. Furthermore, no significant changes were observed in the transcript levels of autophagy related genes [40]. This makes it unlikely that autophagy has been responsible for the observed decrease of the protein levels of ACVS and IPNS during degeneration. In previous studies with the same *P. chrysogenum *strain it has been observed that the rate of penicillin production highly correlates with the IPNS activity [17], whereby a six fold reduction in the specific rate of penicillin production coincided with an approximately six fold decrease of the IPNS activity in glucose limited chemostat cultures. Remarkably, the decrease of the protein levels of ACVS and IPNS during degeneration is much larger than the mean normalized expression of the genes encoding for these proteins, *pcbAB *and *pcbC *(figure 4A). However, a lack of correlation between transcript levels and the levels of the corresponding proteins is a well-known phenomenon [41,42]. A possible explanation for the decreased enzyme levels could be occurrence of point mutations in the ACVS and IPNS genes, resulting in inactive and possibly misfolded proteins which are specifically degraded. It seems, however, unlikely that this would have been caused by mutations during the 10-12 generations cultivation in carbon limited chemostats, considering the speed of degeneration and the fact that the strain contained multiple copies of the penicillin gene cluster. Furthermore impairment of post translational modification could result in the production of non-functional protein. ACVS requires post-translational phosphopantetheinylation to become active [43]. This reaction is catalyzed by a PPTase (4'-phosphopantetheinyl transferase). Inspection of the transcript level of the *P. chrysogenum *PPTase gene (Pc13g04050) during degeneration did, however, not reveal any change in transcript level, indicating that post translational modification of ACVS was not affected.

Several authors have observed that the decrease of penicillin production during prolonged cultivation is accompanied by segregation, that is, the appearance of two or more different phenotypes with lower or no productivity, altered sporulation efficiency, different morphology and increased biomass yield [13,15]. It has been observed, e.g. for oxytetracycline production in an industrial strain of *Streptomyces rimosus *[19], that strain improvement programs could result in high producing strains which are not completely homogeneous, i.e. which contain small amounts of low or non-producing variants. Due to the advantage of the low or non-producing variants under carbon limited conditions they would rapidly outcompete the high producing cells. To verify if this phenomenon could explain the observed degeneration in our high producing strain we, carried out model simulations of carbon limited chemostat growth using a recently published gene regulation model for growth and penicillin production of *P. chrysogenum *[17]. Hereby the maximum biomass and penicillin yields and maintenance energy needs were incorporated which have been determined previously for this strain [16]. Simulations of the initial batch phase and subsequent chemostat phase were carried out, whereby a part of the population was considered to consist of a non-producing variant. These simulations revealed that the observed rapid degeneration in our chemostat cultures could only be described if 15% of the population would consist of a non-producing variant (additional file 1, Figure S16). If the observed degeneration in our cultivations would have been caused by outcompeting of the high producing part of the population by a low producing part, then the vast majority of the degenerated culture would consist of the low or non-producing variant. This would imply that the low or non-producing variant, in spite of containing the full genetic information for penicillin biosynthesis and proper gene transcription, is unable to produce sufficient levels of two key enzymes of the pathway, ACVS and IPNS, possibly due to point mutations. Further investigations are required to elucidate whether culture heterogeneity, as described above, is the cause of the observed degeneration or whether an extremely fast process results in the deterioration of ACVS and IPNS biosynthesis in the producing cells, e.g. trough rapid loss of the translation efficiency or proper posttranslational modification followed by specific degradation of these enzymes.

## Conclusions

A systems biology approach was performed to analyze degeneration of product formation in high penicillin producing cultures of *P. chrysogenum*. To this end, 4 independent ethanol limited chemostat cultivations were carried out at a dilution rate at which the specific penicillin production rate was known to be maximal. We found that the specific penicillin production reached a maximum level between 40 and 80 hours of chemostat cultivation and thereafter decreased more than tenfold during the next 270 hours (approximately 12 generations). This decrease could not be attributed to genetic instability of the penicillin amplicon, neither to down regulation of the penicillin biosynthesis pathway. Nevertheless the protein levels of ACVS and IPNS were found to decrease significantly during degeneration. Also the intracellular levels of the amino acid precursors Val and AAA decreased, but this could well have been caused by the decreased flux towards penicillin. Possible causes for the observed decreased protein levels could be a strong decrease of the translation efficiency of ACVS and IPNS during degeneration or the presence of a culture variant impaired in the biosynthesis of functional proteins of these enzymes, which outcompeted the high producing part of the population.

## Methods

### Strain

Chemostat experiments were performed with the industrial high penicillin production strain *Penicillium chrysogenum *DS17690, kindly donated by DSM Biotechnology Center (Delft, The Netherlands). Chemostat experiments aimed at microbody quantification were performed with a *Penicillium chrysogenum *DS17690 strain which contained green fluorescent protein (GFP) containing the carboxyterminal tripeptide serine lysine leucine that functions as a peroxisomal targeting signal (GFP·SKL) (strain DS58274). This strain was described as DS54465 GFP·SKL before [28] and was kindly donated by W.H. Meijer (University of Groningen, The Netherlands).

### Media and chemostat cultivation

The cultivation medium was prepared as described earlier [9] and contained 0.25 Cmol/L ethanol and 4 mM PAA and was used for the batch and chemostat phase of the fermentations. This medium allowed a steady state biomass concentration of about 3 g DW/L.

Ethanol-limited chemostat cultivations were performed according to Nasution et al. [10] in a 7 L turbine stirred reactor with a working volume of 4 L under aerobic conditions (DO > 70%) and a dilution rate of 0.03 h^-1 ^at 25°C and pH 6.5. Chemostat cultivations were preceded by a batch phase The chemostat feed was started just before ethanol depletion, to prevent starvation of the cells. Chemostat cultivations were carried out for a period of maximally 750 h, representing about 30 generations.

Sampling for transcriptome measurements, Western blotting and metabolome measurements was performed after about 75, 200, 350 and 500 h.

### Continued subcultivation of degenerated culture

After about 500 hours of cultivation (14 generations), 4 mL of degenerated culture was transferred aseptically to a fermentor containing 4 L of fresh cultivation medium, for sub cultivation. The cultivation conditions (batch phase followed by chemostat cultivation) were the same as applied to the parent culture.

### General fermentation analysis

Concentrations of PAA and PenG in the culture supernatant were measured with HPLC-UV [44]. Biomass concentration and offgas oxygen and carbon dioxide analyses were performed as described earlier [9].

### Quantification of the copy number of the penicillin gene cluster

Biomass sampling was performed as described previously [6]. DNA was isolated using a beat-bead procedure using 2-4 glass beads (2.5 mm) to accomplish cell lysis and subsequent DNA purification using the FastDNA^® ^SPIN Kit (MP Biomedicals, Solon, OH). Real-time quantitative PCR was performed in triplicate in 25 μL reaction volume of IQ SybrGreen Supermix (Bio-Rad, Hercules, CA), 0.5 μM of each primer and 2 ng genomic DNA using iQ5-Multicolor Real-Time PCR (Bio-Rad, Hercules, CA, USA). To quantify the number of penicillin gene clusters, *pcbAB *was amplified (forward primer: GGAGCAGGTCTGACGAAGG, reverse primer: AACGAACGGTGTGATATGAACG) together with *niaD *as internal reference (1 genomic copy, forward primer: TGGAGGAAC TGGCATCACAC, reverse primer: ACATAAGCATCAAGGTCAGAACG). The following PCR settings were used: 1 cycle of 95°C for 3 min and cycles of 95°C for 10 s, 58°C for 45 s, 72°C for 45 s and 95°C for 1 min. As a control we used *P. chrysogenum *Wisconsin 54-1255 as it is known to have a single penicillin gene cluster [4,26].

### Transcriptome analysis

Sampling, RNA extraction, microarray analysis, data analysis and clustering were performed as described earlier [6,45]. In short, samples were taken via a sampling port in the wall of the reactor, filtered and immediately quenched in liquid nitrogen. Upon analysis, cells were ground with a mortar and pestle under constant cooling with liquid N_2_. After extraction with trizol and chloroform, RNA was isolated using a phenol-chloroform extraction method. Double stranded cDNA was synthesized from the isolated RNA using a One Cycle cDNA Synthesis Kit (Affymetrix, Santa Clara, USA) and after purification biotinylated cRNA was hybridised to Affymetrix custom-made *Penicillium chrysogenum *GeneChip^® ^microarrays (array code DSM_PENa520255F). Acquisition and quantification of array images were performed using Affymetrix GeneChip Operating Software (GCOS version 1.2) and all expression data were tested against the hypothesis that expression was not significantly different. Differentially expressed genes were enriched in MIPS categories using a Fisher's Exact test. Promoter analysis was performed using the web-based software Multiple Em for Motif Elucidation (MEME). The transcriptome data produced in this study have deposited at the Genome Expression Omnibus database http://www.ncbi.nlm.nih.gov/geo/ under the accession number GSE24212 and are also provided as an excel file (additional file 2).

### Western blotting

Samples for Western blotting were obtained in freeze buffer as described previously [46], precipitated with 12.5% TCA and stored at -20 °C. After thawing on ice, from each sample three independent aliquots were taken and crude protein extracts were prepared as described previously [7]. Protein concentrations were determined using the RC/DC Protein Assay system (Bio-Rad, Hercules, CA,) using bovine serum albumin as standard. Subsequently, western blots were prepared in triplicate utilizing the three independently taken aliquots, with equal amounts of protein loaded per lane - for ACVS, IPNS and AT determinations, 60 μg, 3/30 μg and 30 μg of protein, respectively. SDS/PAGE and Western blotting were performed according to established procedures. Western blots were decorated with specific polyclonal antibodies against ACVS, IAT and IPNS. As secondary antibody anti-rabbit IgG-AP (Santa Cruz biotechnology, Santa Cruz, CA) was used with NBT/BCIP (Roche, Penzberg, Germany) as substrate. After detection, Western blots were scanned using a Bio-Rad GS-710 densitometer. ACVS, IAT and IPNS-specific bands were quantified three times each using ImageJ 1.40 (http://rsbweb.nih.gov/ij/index.html). After averaging, values were expressed as percentages of the initial value, which was set to 100%.

### Microbody quantification

For microbody quantification chemostat experiments were performed with *P. chrysogenum *DS58274 which contained a microbody-targeted green fluorescent protein. These chemostats were performed exactly as all other chemostats with *P. chrysogenum *DS17690.

Biomass from 1.5 mL culture broth samples was fixed using 3.7% formaldehyde in a 50 mM pH 7.2 potassium phosphate buffer on ice for 1 h (centrifugation for 5 min. at 6000 rpm) and stored at 4°C until further analysis. From each hyphae the peroxisomes in two sub apical cells were counted and averaged using a confocal laser scanning microscope (CLSM) as described earlier [28,47]. The number of peroxisomes per cell was determined by counting up to 200 peroxisomes and dividing by the number of cells.

### Metabolome analysis

Sampling and sample processing for intracellular metabolite analysis was performed as described earlier [35]. Quantification was performed as described earlier for metabolites of central metabolism [48], for adenine nucleotides [49] and for free amino acids [50]. Sampling and sample processing for intracellular metabolites related with penicillin biosynthesis including intracellular PAA and PenG levels was performed using a new rapid quenching and filtration based washing technique as described before [11] to efficiently remove the very large extracellular amounts of PAA and PenG.

### Calculation methods

In chemostat cultures with ethanol as sole limiting carbon source, the penicillin concentration will not reach a stable steady-state value, because degeneration is very pronounced. Therefore the biomass specific penicillin production rate q_p _as function of time was calculated from the dynamic penicillin mass balance using polynomial fits for the measured biomass and penicillin concentrations as a function of time:

(1) $$\frac{dC_{p}\left(t\right)}{dt}=q_{p}\left(t\right)\cdot C_{x}\left(t\right)-D\cdot C_{p}\left(t\right)$$

The other specific rates q_s_, q_O2_, q_CO2 _and μ were calculated using mass balances and these rates were used for metabolic flux calculation using the stoichiometric model from van Gulik et al. [9,10] assuming pseudo steady-state.

The reversible reactions from F6P to Mannitol6P and FBP to G3P can be used as a sensor reaction to determine the change in redox status in the cytosol. The relative ratio of NADH to NAD [36] compared to the first time point (t1, high q_p_) can be calculated as follows:

(2) (NADHNAD)t(NADHNAD)t1=(Mannitol6PF6P)t(Mannitol6PF6P)t1

(3) (NADHNAD)t(NADHNAD)t1=(G3PFBP)t(G3PFBP)t1

For the calculation of the extra- to intracellular concentration ratio of PAA the assumed intracellular volume was 2.5 mL/g DW [51,52].

## Authors' contributions

RDD designed the experiments, carried out fermentations, performed sample analysis, drafted the manuscript and coordinated the study, JMB carried out fermentations and performed sample analysis, KMT carried out fermentations, performed sample analysis and quantified microbodies, JAKWK performed Western Blotting, AMK quantified microbodies, ZZ calculated metabolic flux profiles, TV performed a DNA microarray analysis, PK and RALB quantified penicillin gene clusters, JMD performed data analysis on DNA microarrays, JJH designed the experiments, analyzed the data and drafted the manuscript and WMvG designed the experiments, performed the data analysis, drafted the manuscript and coordinated the study. All authors read and approved the final manuscript.

## Supplementary Material

Additional file 1 **Supplementary material I.pdf**. Metabolome data. Click here for file

Additional file 2 **Supplementary material II.xls**. Transcript data of chemostat cultivations 1 and 2. Click here for file
