# Supplementary material for: Degeneration of penicillin production in ethanol-limited chemostat cultivations of Penicillium chrysogenum: A systems biology approach
Source: BMC Syst Biol. 2011 Aug 19;5:132. doi: 10.1186/1752-0509-5-132 (PMC3224390; doi:10.1186/1752-0509-5-132)
Supplement: Additional file 1 — Supplementary material I.pdf. Metabolome data. [file 1752-0509-5-132-S1.PDF]

Supplementary material I

Physiological data continued subcultivation

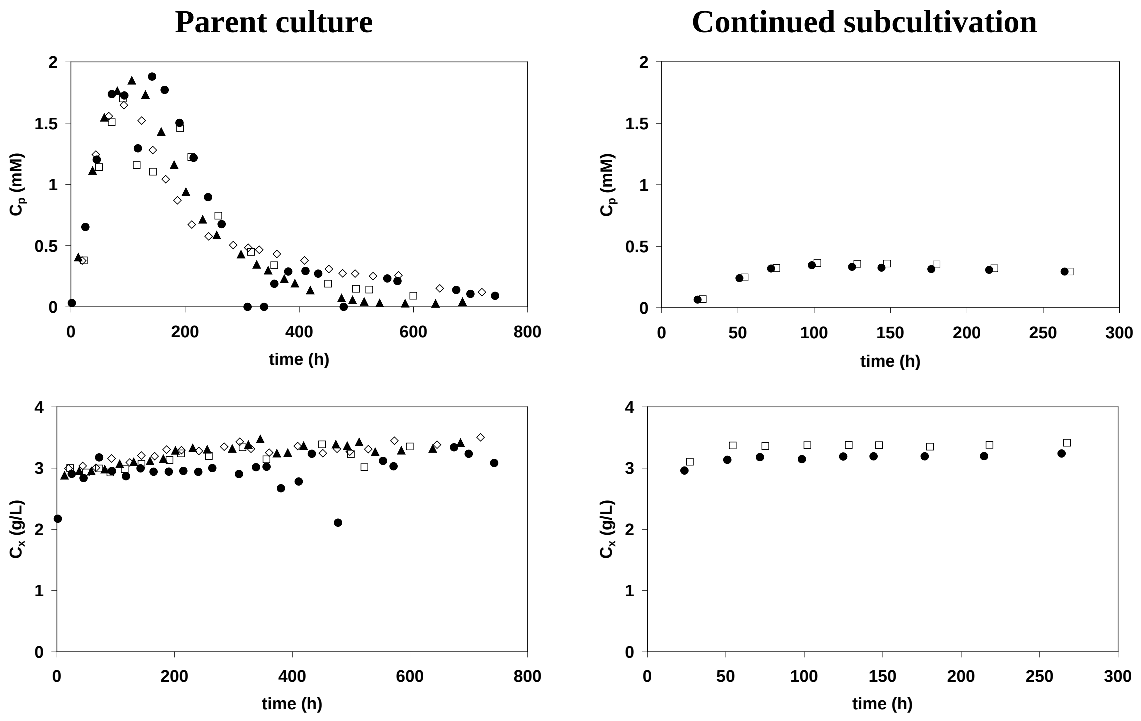

Figure S1 Penicillin concentration in Chemostat 1 (●), 2 (□), 3 (▲) and 4 (◊) (left) and subchemostat 4.1 (●) and 4.2 (□) (right)

Penicillin gene cluster quantification continued subcultivation

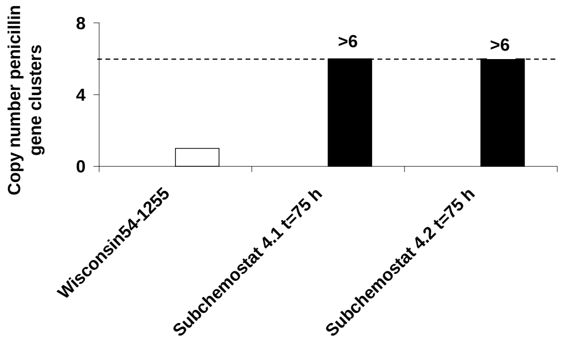

Figure S2 Penicillin gene cluster copy number quantification in the reference strain (Wisconsin 54-1255, 1 penicillin gene cluster, white) and the continued subcultivations 4.1 and 4.2 at the penicillin production peak (t = 75 h, black).

Proteome continued subcultivation

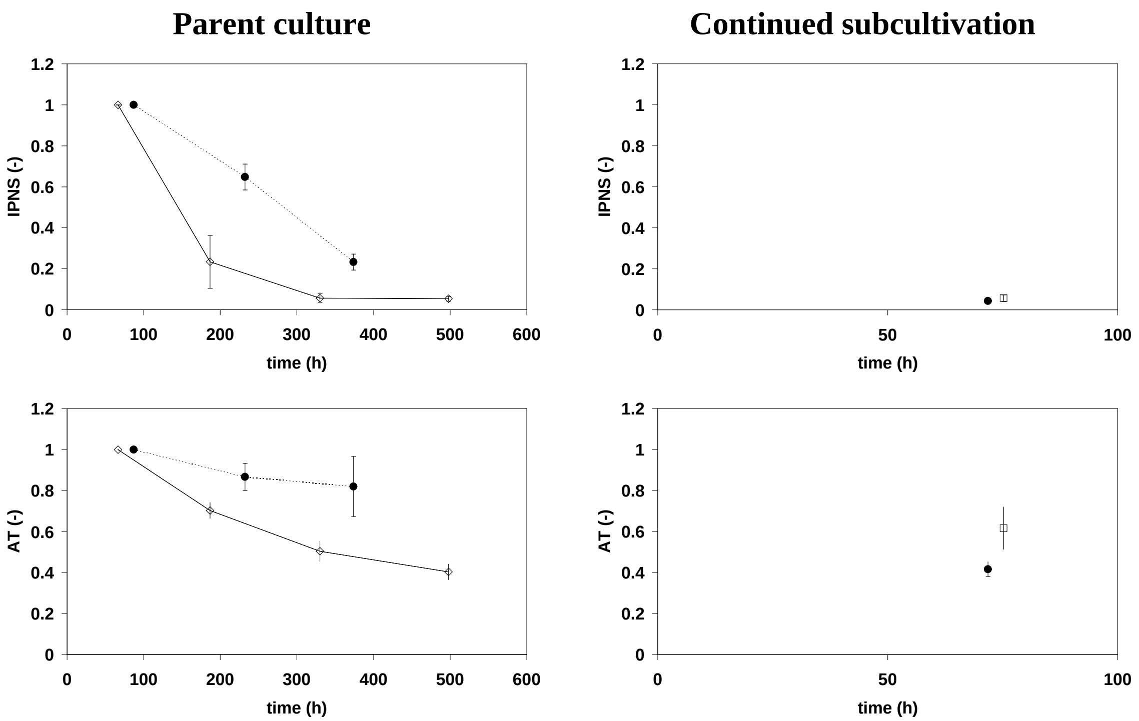

Figure S3 Relative total protein amount of IPNS and AT during chemostat 1 (●) and 4 (◊) (left) and subchemostat 4.1 (●) and 4.2 (□) (right).

Transcriptome

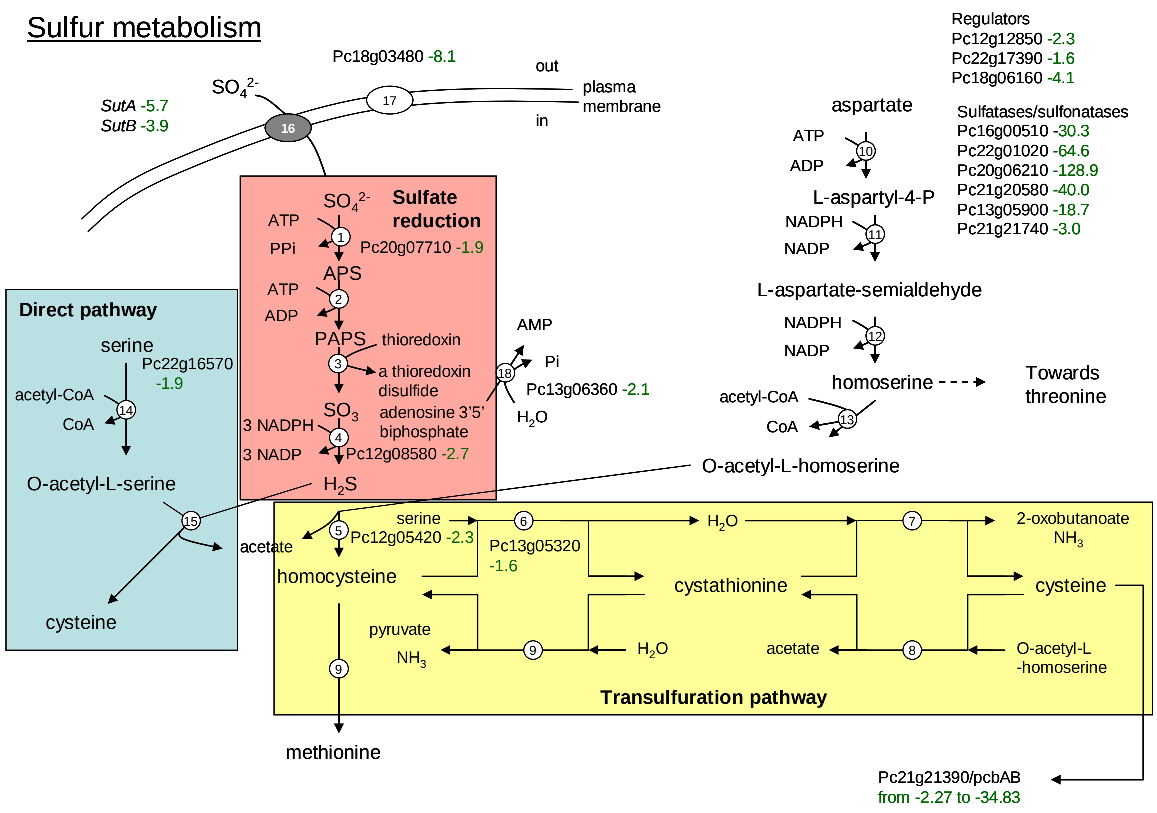

Figure S4 Mean normalized expression of genes in sulfur metabolism in *P. chrysogenum* throughout ethanol limited chemostat cultivation. The numbers behind the transcripts represent the average fold change of chemostat 1 and 3 of expression in the degenerated culture at t = 500 h versus the penicillin production peak at t = 75 h in the prolonged ethanol-limited chemostat run

Metabolome

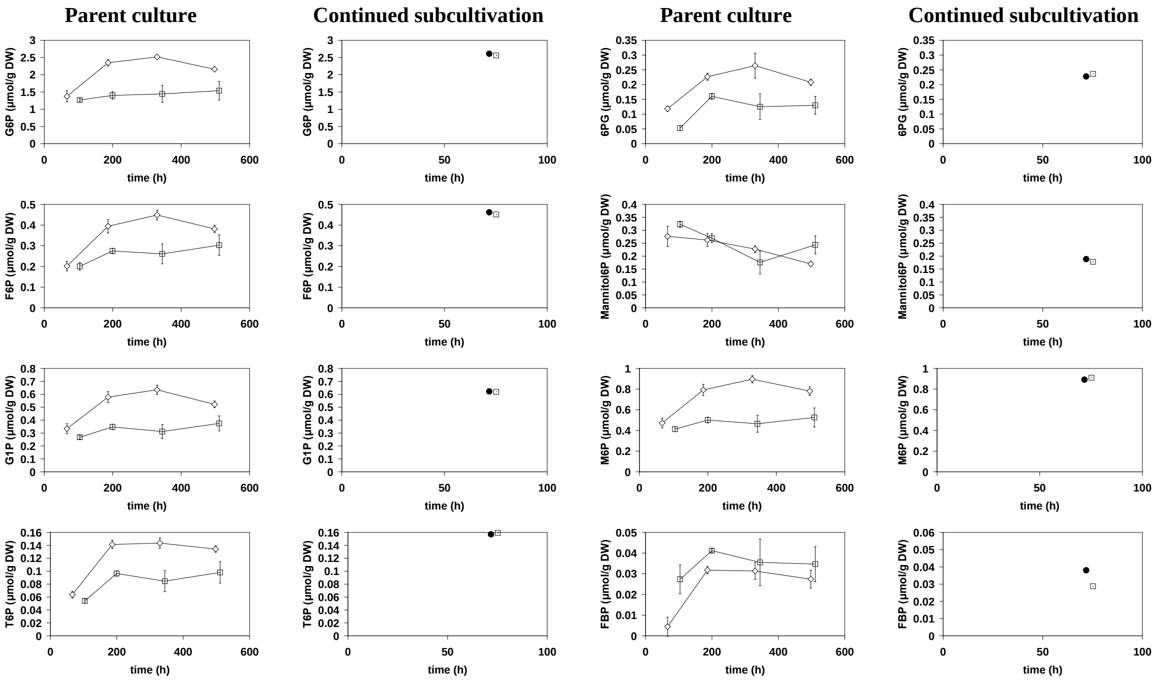

Figure S5 Metabolite amounts of upper gluconeogensis and related metabolites in chemostat 2 (□) and 4 (◊) (left) and subchemostat 4.1 (●) and 4.2 (□) (right).

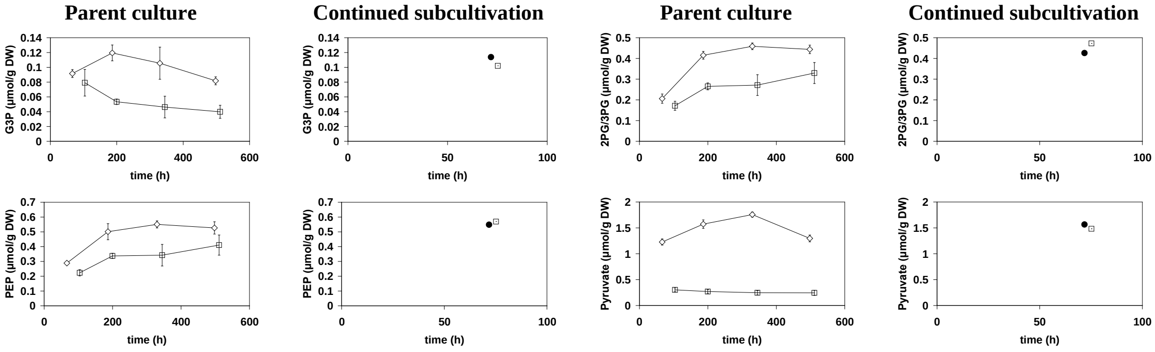

Figure S6 Metabolite amounts of lower gluconeogenesis in chemostat 2 (□) and 4 (◊) (left) and subchemostat 4.1 (●) and 4.2 (□) (right).

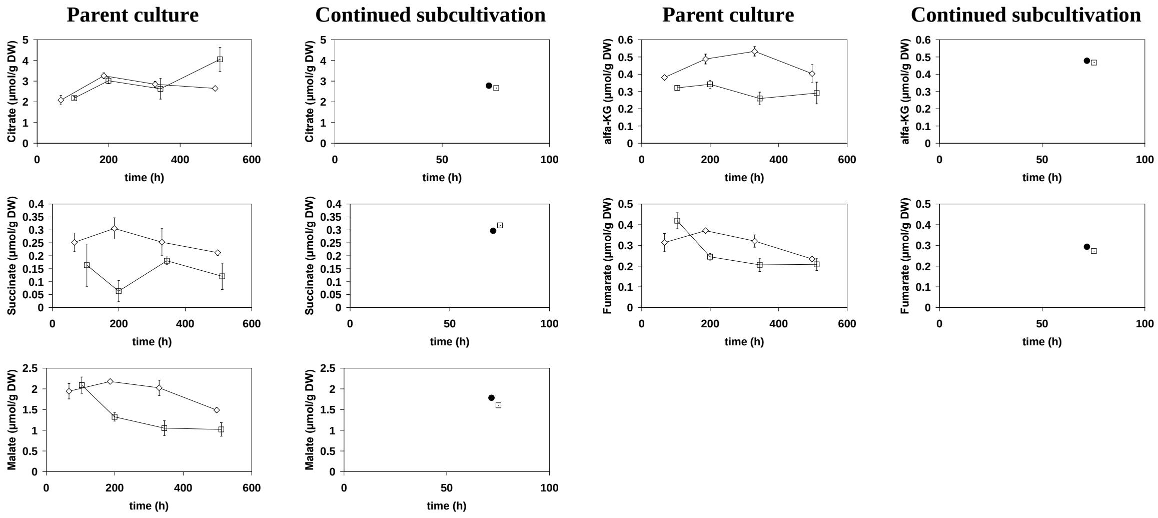

Figure S7 Central metabolite in chemostat 1 (●), 2 (□) and 4 (◊) (left) and subchemostat 4.1 (●) and 4.2 (□) (right).

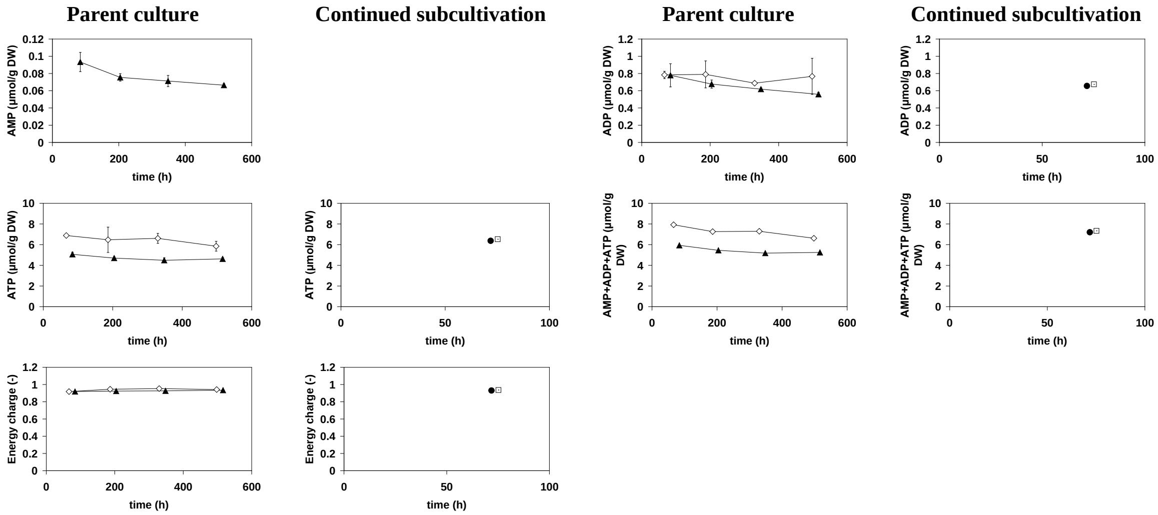

Figure S8 Adenine nucleotides in chemostat 3 (▲) and 4 (◊) (left) and subchemostat 4.1 (●) and 4.2 (□) (right).

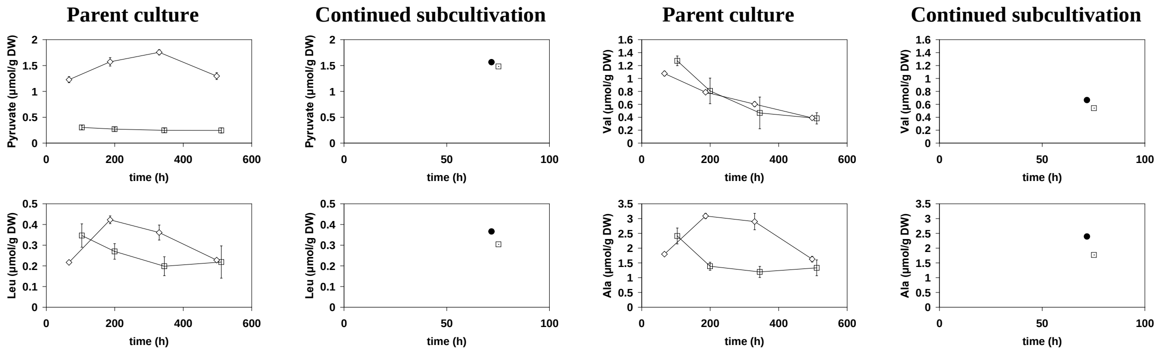

Figure S9 Amino acids from pyruvate in chemostat 2 (□) and 4 (◊) (left) and subchemostat 4.1 (●) and 4.2 (□) (right).

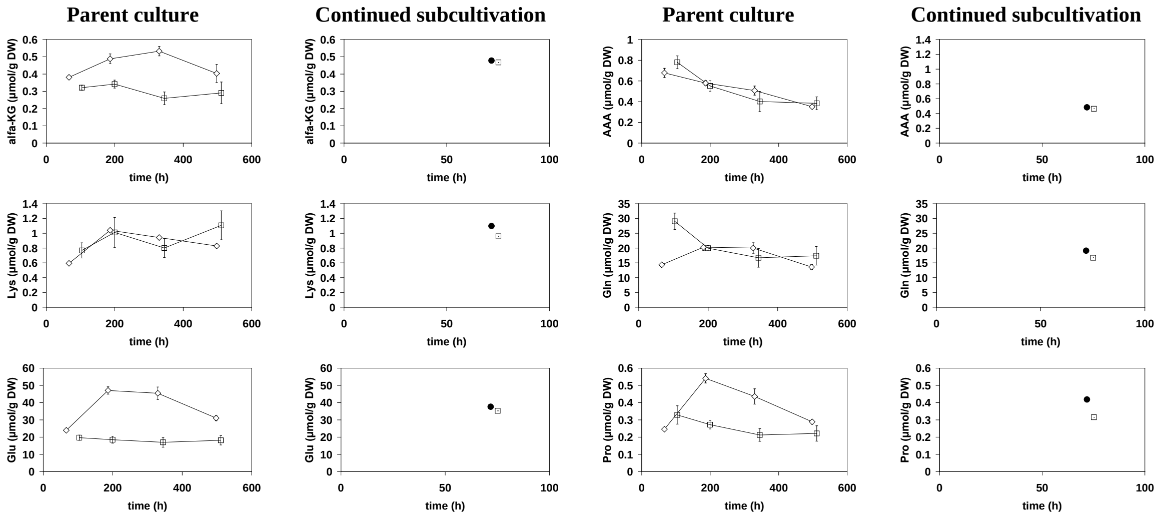

Figure S10 Amino acids from alfa-KG in chemostat 2 (□) and 4 (◊) (left) and subchemostat 4.1 (●) and 4.2 (□) (right).

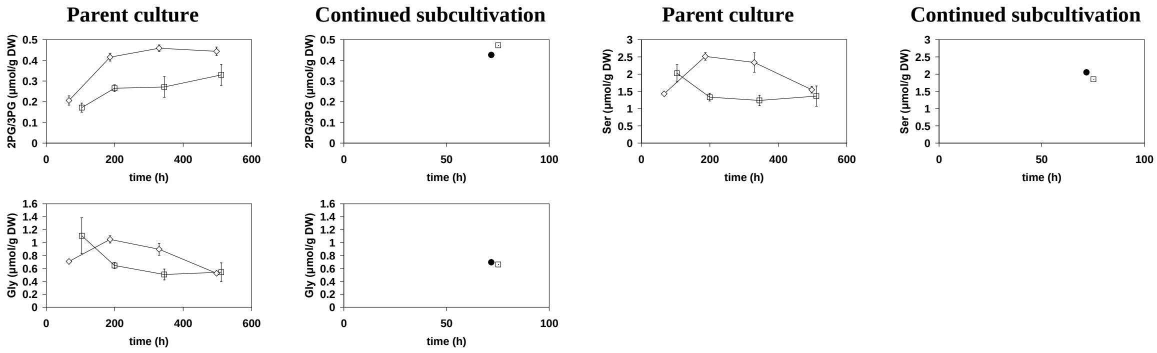

Figure S11 Amino acids from 3PG in chemostat 2 (□) and 4 (◊) (left) and subchemostat 4.1 (●) and 4.2 (□) (right).

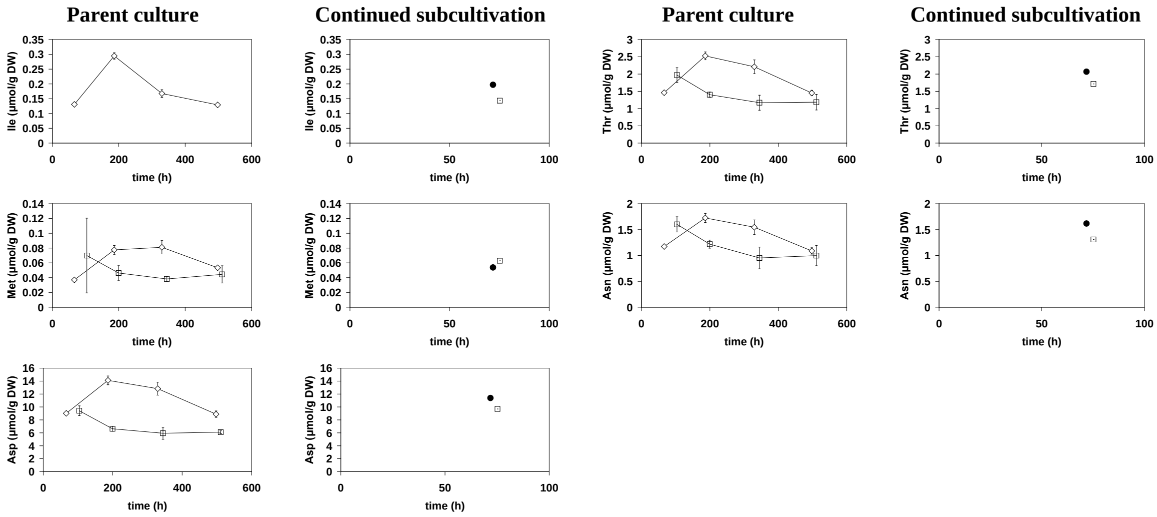

Figure S12 Amino acids from oxaloacetate in chemostat 2 (□) and 4 (◊) (left) and subchemostat 4.1 (●) and 4.2 (□) (right).

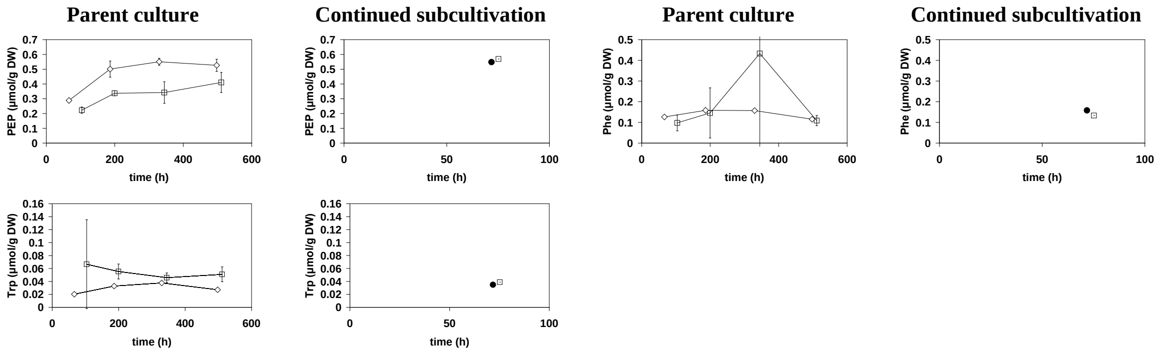

Figure S13 Amino acids from E4P and PEP in chemostat 2 (□) and 4 (◊) (left) and subchemostat 4.1 (●) and 4.2 (□) (right).

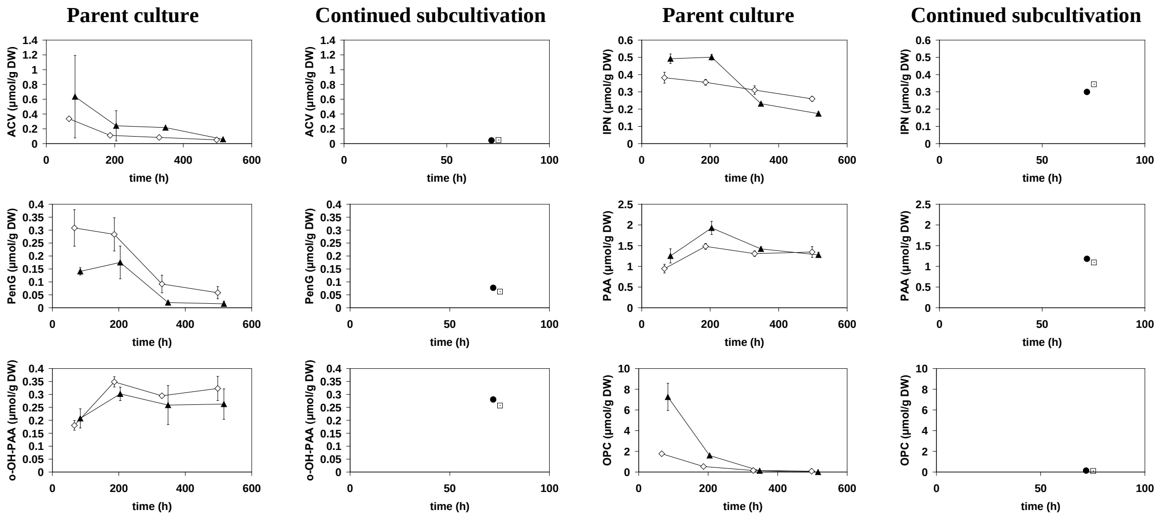

Figure S14 Metabolites related with penicillin biosynthesis during chemostat 3 (▲) and 4 (◊) (left) and subchemostat 4.1 (●) and 4.2 (□) (right).

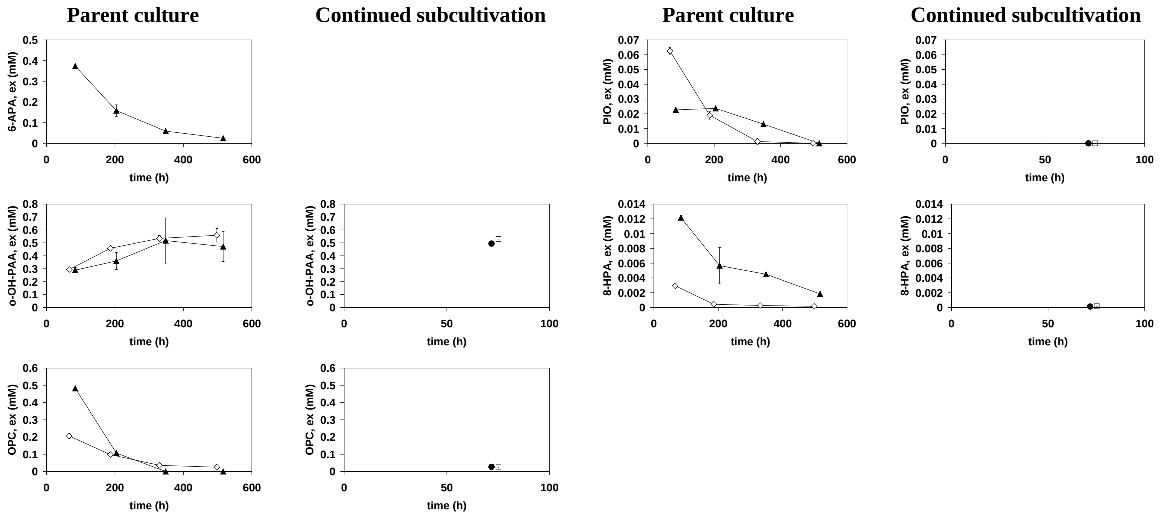

Figure S15 Extracellular levels of metabolites related with penicillin biosynthesis during chemostat 3 (▲) and 4 (◊) (left) and subchemostat 4.1 (●) and 4.2 (□) (right).

Population simulations

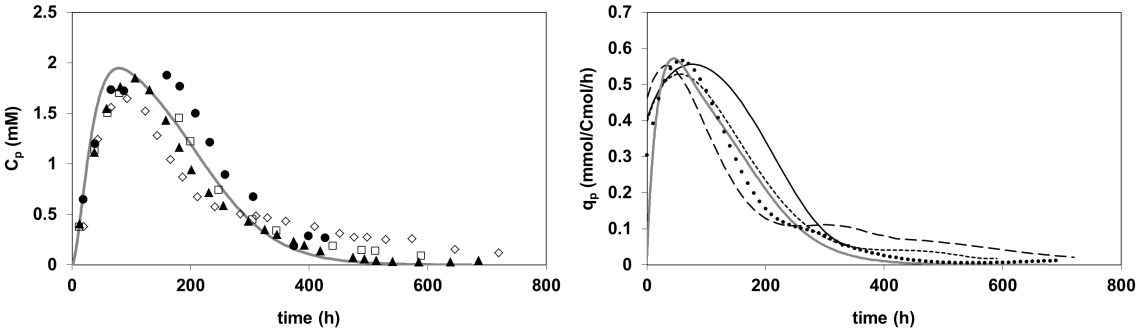

Figure S16. Measured and simulated (grey lines) time courses of PenG concentration and specific PenG production rate during growth in ethanol limited chemostat at a dilution rate of 0.03 h^{-1}. Simulations were carried out using the gene regulation model model published by Douma et al. [17] whereby 15% of the initial population was assumed to consist of non-producing cells.
